# *xrd_simulator*: 3D X-ray diffraction simulation software supporting 3D polycrystalline microstructure morphology descriptions

**DOI:** 10.1107/S1600576722011001

**Published:** 2023-02-01

**Authors:** Axel Henningsson, Stephen A. Hall

**Affiliations:** a Div. Solid Mechanics, Lund University, Ole Römers väg 1, Lund, Sweden; Ecole National Supérieure des Mines, Saint-Etienne, France

**Keywords:** X-ray diffraction, 3DXRD, simulation tools, polycrystalline microstructure, computer programs

## Abstract

An open source Python package named *xrd_simulator*, capable of simulating geometrical interactions between a monochromatic X-ray beam and a polycrystalline microstructure, is described and demonstrated. The software can simulate arbitrary intragranular lattice variations of single crystals embedded within a multiphase 3D aggregate by making use of a tetrahedral mesh representation where each element holds an independent lattice. By approximating the X-ray beam as an arbitrary convex polyhedral region in space and letting the sample be moved continuously through arbitrary rigid motions, data from standard and non-standard measurement sequences can be simulated. This implementation is made possible through analytical solutions to a modified, time-dependent version of the Laue equations. The software, which primarily targets three-dimensional X-ray diffraction microscopy (high-energy X-ray diffraction microscopy) type experiments, enables the numerical exploration of which sample quantities can and cannot be reconstructed for a given acquisition scheme. Similarly, *xrd_simulator* targets investigations of different measurement sequences in relation to optimizing both experimental run times and sampling.

## Abstract 2

An open source Python package named *xrd_simulator*, developed to address the need for 3D microstructure morphology simulation in 3DXRD-type experiments, is described and demonstrated.

## 1. Introduction

Three-dimensional X-ray diffraction (3DXRD) covers a class of experimental techniques that facilitate the nondestructive study of polycrystalline materials on an inter- and intra-granular level. In its original form, 3DXRD, which is sometimes referred to as high-energy X-ray diffraction microscopy (HEDM) (Bernier *et al.*, 2020 ▸), was pioneered by Poulsen (2004 ▸) and co workers. The data for 3DXRD are acquired using monochromatic, parallel, hard X-ray beams (10–100 keV) and a 2D area detector that integrates the diffraction signal from a rotating polycrystalline sample. The samples typically studied using 3DXRD, in contrast to those studied with powder diffraction techniques, are polycrystals with a limited number of grains, allowing individual diffraction peaks to be resolved on the 2D detector image. The recorded diffraction peaks can be analysed using a plethora of methods to reconstruct, among other things, grain orientations (Lauridsen *et al.*, 2001 ▸; Sharma *et al.*, 2012*a* ▸,*b* ▸), grain topology (Poulsen & Schmidt, 2003 ▸; Poulsen & Fu, 2003 ▸; Alpers *et al.*, 2006 ▸; Batenburg *et al.*, 2010 ▸), and grain strain or stress tensors (Oddershede *et al.*, 2010 ▸). The beam cross section and angular step size in 3DXRD must be selected such that a limited number of grains are illuminated during detector readout, limiting spot overlap and revealing the individual diffraction peaks from grains within the aggregate in the 2D detector images. 3DXRD geometries using a narrow beam cross section, smaller than the grain diameter, are often referred to as scanning-3DXRD (Hayashi *et al.*, 2015 ▸). These methods allow for the study of intragranular effects (Hayashi *et al.*, 2017 ▸; Hektor *et al.*, 2019 ▸; Henningsson *et al.*, 2020 ▸) at the cost of having to scan the sample across the narrow beam to collect the full diffraction signal. Another branch of 3DXRD is diffraction contrast tomography (DCT) (Ludwig *et al.*, 2009 ▸), where the detector is placed close to the sample such that the projection of individual grain shapes can be seen in the recorded diffraction image. Using iterative reconstruction methods [*e.g.* Reischig & Ludwig (2020 ▸)] in conjunction with DCT methods, excellent resolution of the grain shapes can be achieved at the cost of strain resolution (Nervo *et al.*, 2014 ▸). For an in-depth summary of the state of the art in hard X-ray microscopy see Poulsen (2020 ▸).

In all of the aforementioned 3DXRD methods, to reconstruct the sample it is necessary to model the sample on a granular or even intragranular level, which stands in contrast to powder-like diffraction experiments where the sample is treated as a continuum. To produce a diffraction pattern of sufficient quality to reconstruct the desired sample details requires selection of experimental parameters such as sample rotation axis, sample translations, X-ray beam shape, detector geometry and sample rotation sequence adapted to the position, shape, orientation and strain of the individual crystals within the polycrystalline aggregate to be studied. The interactions between these acquisition and sample characteristics regulate the quality/resolution of the reconstructions of the sample microstructure as well as the total acquisition times, which can become unrealistically long. The question as to how measurements should be acquired and how many acquisitions are needed to recover a target quantity in a polycrystal are, thus, key in the field of 3DXRD. For instance, by analytical means, Lionheart & Withers (2015 ▸) showed that the full strain tensor could be recovered using direct methods if the diffracting sample was allowed to rotate consecutively around three orthogonal axes. On the other hand, using mechanical constraints, it was found that strain reconstructions could be achieved from single axis rotation data (Henningsson & Hendriks, 2021 ▸). On another note, recent advances in acquisition strategies for laboratory-based DCT (Oddershede *et al.*, 2022 ▸) suggest that more complex scan geometries could be used to improve sampling in 3DXRD experiments. From a practical point of view, considering scanning 3DXRD, the typical wall times to measure a single sample volume are often in the range of hours or even days [*e.g.* Hektor *et al.* (2019 ▸)], making efficient measurement schemes that can reduce the amount of data that need to be collected attractive.

As 3DXRD is a high-energy synchrotron technique, access to experiments is precious and the number of facilities in the world that offer 3DXRD controls the pace of the method development. An alternative route for development is the use of software simulation tools that can serve as a research primer, allowing ideas to be established or discarded at a theoretical stage. Many tools for simulating X-ray diffraction from individual crystals exist [*e.g.* Macrae *et al.* (2006 ▸), Momma & Izumi (2008 ▸), Soyer (1996 ▸), Campbell (1995 ▸), Huang (2010 ▸), Kanagasabapathy (2016 ▸), Weber (1997 ▸) and Laugier & Bochu (2001 ▸)]. Additional tools exist for simulating 2D diffraction patterns from arbitrarily textured samples (Poulsen, 2004 ▸; Le Page & Gabe, 1979 ▸; E *et al.*, 2018 ▸; Huang *et al.*, 2021*a* ▸,*b* ▸; Knudsen, 2009 ▸; Bernier *et al.*, 2011 ▸; Pagan *et al.*, 2020 ▸; Fang *et al.*, 2020 ▸; Sørensen *et al.*, 2012 ▸). However, for many questions related to 3DXRD techniques, the geometry of the polycrystal grains and the X-ray beam, together with intragranular lattice variations, must be accounted for. At the same time, the diffracting sample must be allowed to move along an arbitrary rigid body motion path, to explore different scan sequences.

Frameworks similar to those developed by Wong *et al.* (2013 ▸) and Song *et al.* (2008 ▸) provide important contributions in this direction, incorporating a spatial description of the sample microstructure by making use of a tetrahedral mesh representation. However, this previous work was limited to full-field illumination and sample motions derived from rotations about a fixed axis. Finite beam sizes, illuminating a subvolume of the samples during diffraction, is especially important to simulate scanning 3DXRD were the beam cross section is smaller than the sample.

In conclusion, no open source software exists with the set of capabilities needed to freely explore acquisition strategies in 3DXRD [see supplementary material of Huang *et al.* (2021*b* ▸) for a useful summary of existing software capabilities].

We report on the development of new software, named *xrd_simulator*, that draws on concepts described by Fang *et al.* (2020 ▸) and extends the work of Wong *et al.* (2013 ▸), to take the beam geometry, the grain shapes and intragranular lattice variations into account using a tetrahedral mesh representation. Additionally, we derive analytical solutions to the Laue equations to calculate the diffraction volumes and vectors for arbitrary positions and orientations of the sample. This enables simulation of diffraction as the sample undergoes user-specified rigid body motion sequences during diffraction readout and can be viewed as a generalization of the equations provided by Wong *et al.* (2013 ▸) for single-axis rotation. By making *xrd_simulator* open source and easily accessible, we provide a means to accelerate the rate at which 3DXRD-type methodologies can evolve.

The paper is structured as follows. In Section 2 we present the diffraction approximations made in *xrd_simulator* and derive the analytical expressions needed for its implementation. In Sections 3 and 4 we comment on the software architecture and availability and provide references to external tutorials and documentation. In Section 5 we comment on the computational aspects of the software and provide sample benchmarks. Finally, in Section 6 we provide some concluding remarks. Additionally, we append a case study comparison of simulations performed with *xrd_simulator* and data collected at the ESRF ID11 beamline.

## 2. Diffraction approximations

X-ray diffraction is computed in *xrd_simulator* by defining a series of mathematical model components, including a polycrystal, an X-ray beam and a detector. In this section we describe the formulation of these models and discuss their interactions. In the following, any vector **v** is normalized by the inclusion of a symbol 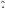 such that 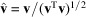 .

**Figure 1:**
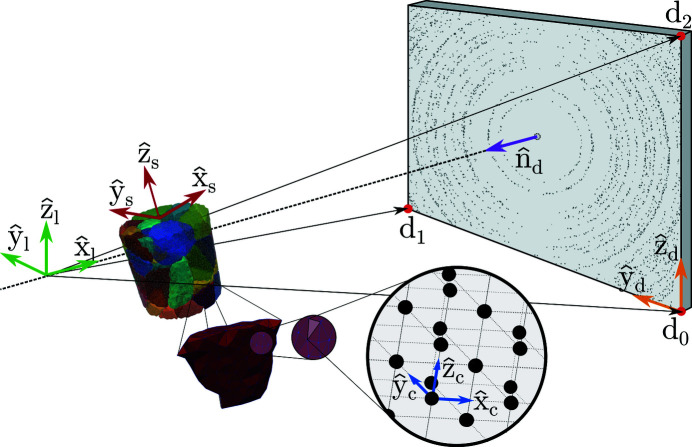
Illustration of *xrd_simulator* laboratory (subscript l), sample (subscript s), crystal (subscript c) and detector (subscript d) coordinates systems. The three corners of the detector (**d**_0_, **d**_1_, **d**_2_) define its position and orientation in space.

Four Cartesian coordinate systems are used; the laboratory coordinate system, the sample coordinate system, the crystal coordinate system and the detector coordinate system (Fig. 1 ▸). The crystal, sample and detector coordinate systems are all fixed in relation to a lattice, a polycrystalline sample and a detector plane, respectively. Transformations of these three coordinates systems are tracked by the laboratory coordinate system, which serves as a global frame of reference.

The morphology of a polycrystalline sample is defined in the global laboratory reference frame with axes 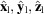 . As a starting point, the internal sample coordinate system, with axes 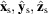 , is aligned with the laboratory system. Once the sample has moved, to transform a point **p**_l_ from laboratory to sample coordinates we apply a rigid body motion through a rotation matrix, **R**, and a translation vector, Δ**x** as 

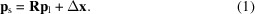

The single crystal elements constituting a polycrystalline sample each have their own crystal coordinate reference frame with axes 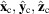 . A vector, **p**_c_, described in crystal coordinates is transformed to the sample frame via the crystal orientation matrix, **U**, as 

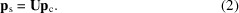

The detector coordinate system, with in-plane axes 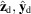 and normal 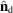 , defines the plane at which a diffraction pattern can be collected. A point on the detector surface, **p**_l_, can be described by its projection onto the in-plane detector axes 

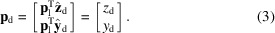




### 2.1. Diffraction equations

We define an incident wavevector, **k**, to point in the propagation direction of a parallel monochromatic X-ray beam. The diffraction vector, **G**, is defined as 

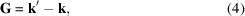

where **k**′ is an elastically scattered wavevector. The Euclidean norm, || · ||, of the wavevector is defined as 

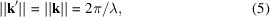

where λ is the X-ray wavelength.

From equation (4) and the elastic scattering condition it follows that 

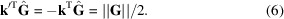

Considering equation (6) together with equation (5), it follows that **k** and −**k**′ form the same angle, π/2 − θ, to **G**. The Bragg angle, θ, can be found as 

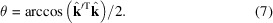

For diffraction to occur from a set of lattice planes the Laue equations require that 

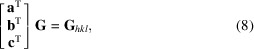

where **a**, **b** and **c** define a unit cell and **G**_*hkl*_ = [*h* *k* *l *]^T^ holds the integer Miller indices of the diffracting lattice plane family. Introducing the unique multiplicative decomposition of the inverse matrix [**a** **b** **c**]^−T^ into a unitary rotation matrix, **U**, and an upper triangular matrix, **B**, with positive diagonal elements, we write equation (8) as 

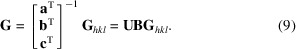

In this description **U** is the crystal lattice orientation matrix while **B** is defined from the lattice unit cell.

### 2.2. Polycrystalline sample representation

**Figure 2:**
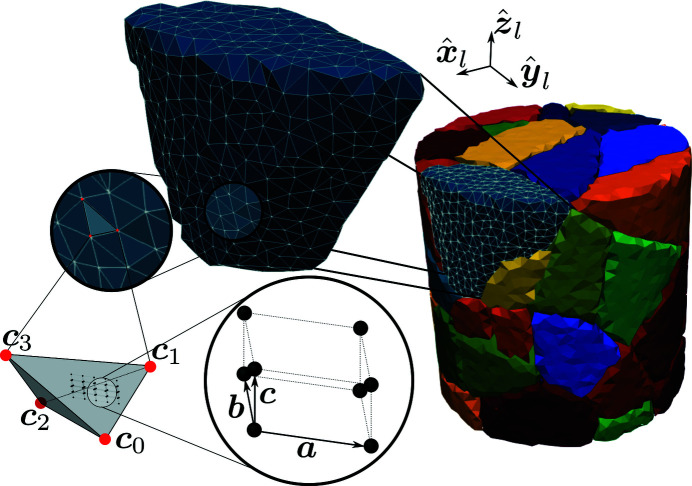
Illustration of a polycrystal representation in *xrd_simulator*. The tetrahedral single crystal elements form a mesh, representing a polycrystalline aggregate. Each individual tetrahedron can hold a unique lattice and phase.

A polycrystalline sample is represented by a tetrahedral mesh with each individual tetrahedron being modelled as a single crystal; grains are thus defined by adjacent cells with the same (or similar) unit-cell parameters (Fig. 2 ▸). The single crystal elements are defined through a reference unit cell, a phase, a symmetric infinitesimal strain tensor (laboratory coordinates), **ε**_*l*_, and a crystal orientation matrix, **U**. Each of these four quantities remain constant over each element volume and spatial variations in the lattice structure are modelled by letting neighbouring elements hold different lattice states. The nodal vertices of a tetrahedron are denoted (**c**_0_, **c**_1_, **c**_2_, **c**_2_), as illustrated in Fig. 2 ▸.

To compute the **B** matrix, given the quantities associated with a single tetrahedron for use in equation (9), we use *xfab*, which is part of the *3DXRD Fable* suite (Sørensen *et al.*, 2012 ▸).

### 2.3. Beam representation

**Figure 3:**
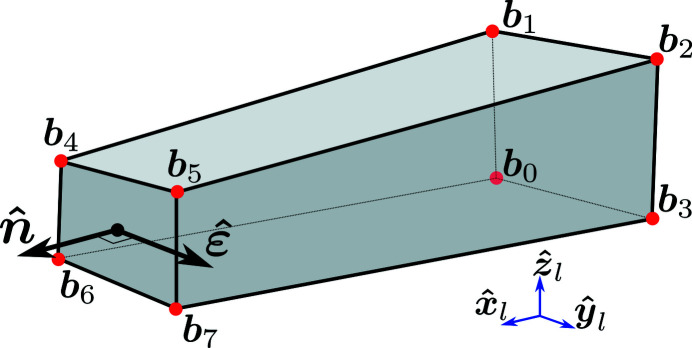
Example of a possible X-ray beam geometry with a total of eight nodes, **b**_*i*_, forming a convex hull in 3D space. Photons propagate in the direction of 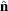 and are linearly polarized along 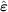 . The photon intensity inside the beam hull is uniform.

A beam of X-rays is represented by a convex polyhedron with *n* vertices, **b**_*i*_, indexed as *i* = 0, 1,…, *n*. The X-ray propagation direction is defined by the unit vector 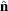 . The photon density is taken to be uniform within the beam hull and the X-rays are assumed to be linearly polarized in the direction of a unit vector, 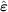 . An example geometry of an X-ray beam is illustrated in Fig. 3 ▸.

The use of a convex polyhedron to represent the beam shape, as opposed to an axis-aligned box for instance, is motivated by the need for *xrd_simulator* to facilitate numerical investigations of scan sequences in far-field X-ray diffraction. Optimal selection of beam cross section shape and scan pattern remain open research questions in scanning 3DXRD experiments. Moreover, the use of a convex beam allows indirectly for simulations of variable beam intensity profiles. This can be achieved by repeatedly computing diffraction from sub regions of a composite beam, one diffraction pattern at a time, to produce a weighted sum of diffraction.

### 2.4. Scattering unit

**Figure 4:**
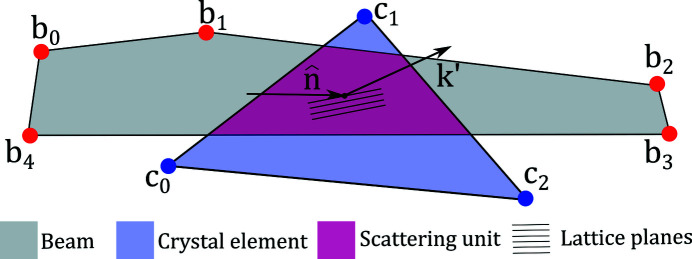
A simplified 2D example of a scattering unit formed as the intersection between the X-ray beam and a single crystal element. Note that *xrd_simulator* uses 3D representations for both beam and crystals.

The volume intersection between an illuminated diffracting single crystal element and the beam is defined as a scattering unit. As both the beam and the single crystal tetrahedrons are convex, their intersections will also form convex polyhedrons. The scattering units each have a diffracted wavevector **k**′ and serve as the basis for rendering diffraction patterns onto the detector area. A simplified 2D illustration of a scattering unit is given in Fig. 4 ▸

To compute the scattering unit polyhedron we use the *SciPy* (Virtanen *et al.*, 2020 ▸) wrapper for the *Qhull* (Barber *et al.*, 1996 ▸) library. The algorithm is seeded with an interior point of the scattering unit polyhedron, which can be found either by trial and error or by solving a linear program, as described in the scipy.spatial.HalfspaceIntersection documentation. Since the computation of the scattering unit polyhedron is expensive, *xrd_simulator* implements a collision detection algorithm that checks for intersections between element bounding spheres and the beam hull. This allows *xrd_simulator* to quickly exclude elements of the mesh that cannot take part in diffraction.

### 2.5. Detector representation

A detector is represented by an arbitrary rectangular plane segment holding a grid of rectangular pixels with user specified size ( 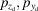 ). As depicted in Fig. 1 ▸, the detector can be parameterized by three vectors (**d**_0_, **d**_1_, **d**_2_) extending from the laboratory origin to the detector corners. The three detector corners are arranged in clockwise order, with respect to the detector normal, and the detector coordinate system origin is taken as **d**_0_. Since the detector corners **d**_0_, **d**_1_ and **d**_2_ may be arbitrarily specified in 3D space it is possible to simulate arbitrary detector tilts and misalignments in *xrd_simulator*. The detector coordinate axes are defined as 

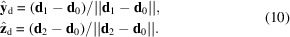

The detector normal is defined through the cross product, 

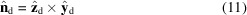

Additionally, a point spread function, PSF(*z*_*d*_, *y*_*d*_), simulating blurring due to the detector optics can be specified. When computing the simulated diffraction data the point spread function is convoluted with the 2D diffraction image, as a final step.

### 2.6. Sample motion

Before the derivation of diffraction vectors can be considered, we must first describe the motion path of the sample during detector readout. An arbitrary rigid body motion of the sample is defined by a unit rotation axis, 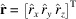 , a rotation angle, Δω ∈ (0, π), and a translation vector, Δ**x**. The motion is executed over the unitless time interval *t* ∈ [0, 1] during which a single detector frame is collected. At the start of detector readout, before the sample has moved, *t* = 0, and at the end of readout, when the sample has translated by Δ**x** and moved Δω radians around 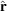 , *t* = 1. In this way, arbitrary scan sequences can be modelled using different sample motions for each detector frame readout.

The sample is modelled to move uniformly over *t* ∈ [0, 1] such that at some intervening time, 0 < *t* < 1, the coordinates of a node, **c**_*i*_ = **c**_*i*_(*t*), in the sample mesh can be found as 

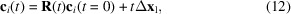


**R** is a Rodriguez rotation matrix, defined as 

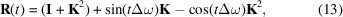

with unity matrix **I**, and 

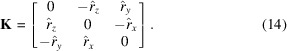

With the motion path of the sample defined through equations (12), (13) and (14), we may now proceed to compute diffraction vectors.

### 2.7. Diffraction computation

By the introduction of arbitrary rigid body motions of the sample in equation (12), the Laue equation (9) becomes time dependent. Solutions to these equations for a fixed rotation axis and no sample translations have been derived by Wong *et al.* (2013 ▸). In the following we generalize these results to facilitate an arbitrary axis of rotation as well as an arbitrary sample translation.

Considering a single crystal element, equations (9) and (13) yield the scattering condition at time *t* as 

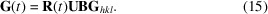

By finding solutions to equation (15) over *t* ∈ [0, 1], the position of the crystal element nodes at the times when diffraction from the volume element can occur can be established through equation (12) together with the diffracted wavevector equation (4). This information defines the scattering unit. The lack of solutions to equation (15) over *t* ∈ [0, 1] means that the crystal cannot diffract over the given sample motion.

To derive solutions to equation (12) in *t* we start by introducing a scalar form of the Laue condition. From equation (6) it follows that 

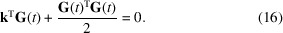

Introducing **G**_0_ = **U** **B** **G**_*hkl*_ and combining equation (13) with equation (16) we find 

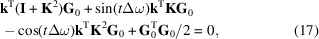

where we use the fact that **G** ^T^(*t*)**G**(*t*) = **G**_0_ **G**_0_ since **R**(*t*) is unitary. Introducing the scalars 

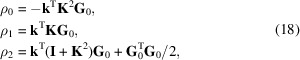

we may write equation (17) as 

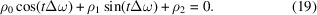

Introducing the variable 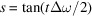 we find from the double-angle formula that 

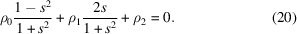

Since equation (20) is a scalar quadratic equation, one, two or zero solutions must exist. Solving for *s* when ρ_2_ ≠ ρ_0_ we find that 

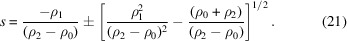

In the special case of ρ_2_ = ρ_0_ equation (20) reduces to 

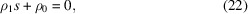

such that a single solution, *s* = −ρ_0_/ρ_1_, can be found, given that ρ_1_ ≠ 0. Finally, the sought time, *t*, in equation (15) is found by reversing the tangent substitution, 

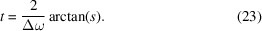

We remind the reader that the derived solutions, *t*, are the relative moments in time, during a frame acquisition, at which a single crystal element will diffract the incident X-rays. The position of the element nodes during diffraction can, thus, be computed through equation (12) and the geometry of the scattering unit is found by computing the intersection of the updated tetrahedral element and the X-ray beam. With this information available we may proceed to propagate the diffracted X-rays onto the 2D detector area.

### 2.8. Ray tracing

**Figure 5:**
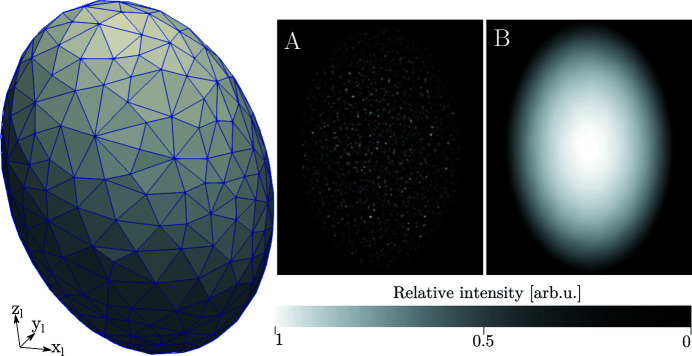
Illustration of a single simulated diffraction peak (right) for an elliptical grain meshed by 3283 elements (left). The difference between ray tracing driven by the scattering unit centroids (A) can be compared with ray tracing driven by the detector pixels grid (B).

Once the scattering units have been established, the diffracted wavevectors, **k**′, are traced onto the detector surface. Two options for ray tracing are available in *xrd_simulator*. Either rays are traced from the centroids of the individual scattering units or, alternatively, rays are traced from the detector pixel centroids back through the scattering units. The latter of the two models can be considered to produce a more accurate projection approximation while the former will be computationally faster. As illustrated in Fig. 5 ▸, ray tracing driven by the detector grid pixels will produce space-filling projections, while ray tracing driven by the scattering unit centroid will approximate a diffraction peak as a point cloud.

Considering a point **x** in the sample volume associated with a scattered wavevector **k**′, we may parameterize a scattered ray through a scalar *h* as 

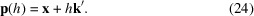

The point of intersection, **p**(*h* ^*^), between scattered ray and detector is found from 

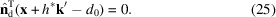

Solving equation (25) for 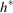 yields 

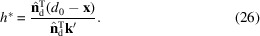

The detector coordinates of the intersection point can now be found through equation (10), 

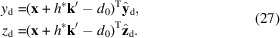

By setting **x** in equation (27) as the scattering unit centroid, ray tracing can be performed. When ray tracing using the detector pixels as source points is considered instead, **x** in equation (24) must be taken as a point in the detector plane. By solving equation (24) for the intersections with the planes that define the facets of the scattering unit, an intersection length, *l*, between the ray and polyhedron can be established. To do so, we have implemented the clipping algorithm developed by Cyrus & Beck (1978 ▸). To speed up the computations, the vertices of a scattering unit are first projected onto the detector plane, establishing a feasible region on the detector where the projection may fall. In this way equation (24) is only solved for a sub-grid of the detector.

### 2.9. Intensity model

Once the diffracted rays of a scattering unit have been mapped to the pixels of the detector, the scattered intensity, *I*, can be computed and deposited. If ray tracing based on the scattering unit centroids is used, the intensity is modelled to be proportional to the scattering unit volume, *V*, polarization factor, *P*, Lorentz factor, *L*, and structure factor, *F*_*hkl*_, as 

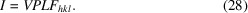

If, instead, ray tracing is driven by the detector pixels, the intensity is modelled as 

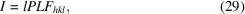

where *l* is the intersection length between the scattered ray and the scattering unit polyhedron.

The inclusion of the factors *P*, *L* and *F*_*hkl*_ in the intensity model of *xrd_simulator* can be toggled by the user. Since *xrd_simulator* is designed to separate the computation of scattering units from the diffraction pattern image rendering, several different intensity and ray tracing combinations can be tested without having to solve equation (15) repeatedly. It is also possible to access the scattering units directly in *xrd_simulator*, allowing for custom intensity and ray tracing models to be tested.

#### 2.9.1. Structure factors

To compute structure factors we use the open source tool *xfab*, which is available as part of the *FABLE-3DXRD* software suite (Sørensen *et al.*, 2012 ▸; https:// github.com/FABLE-3DXRD/xfab). An introduction to structure factors is provided by, for example, Als-Nielsen & McMorrow (2011 ▸). To include structure factors in the intensity model the user is expected to provide a crystallographic information file (Hall *et al.*, 1991 ▸) to *xrd_simulator*, specifying the properties of the simulated material phases. If structure factors are not needed, the user may alternatively define the material phase by passing a set of unit-cell parameters.

#### 2.9.2. Lorentz factors

As stated by Lauridsen *et al.* (2001 ▸), for a single axis rotation geometry, where the rotation axis is aligned with 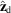 , the Lorentz factor can be approximated as 

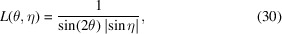

where η denotes the angle between the projection of the rotation axis, 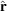 , and scattered ray direction, 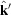 , onto the 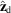 – 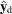 plane. In *xrd_simulator* each detector frame has an arbitrary sample rotation axis and η can be found as 

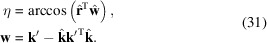

By additionally recovering θ from equation (7), the Lorentz factor can be computed from equation (30). Note that the expression for the Lorentz factor in equation (30) is approximate. Especially, for η = 0 or θ = 0, the intensity will diverge, and *xrd_simulator* will insert numpy.inf values at the corresponding detector pixels.

#### 2.9.3. Polarization factors

For linearly polarized X-rays (Als-Nielsen & McMorrow, 2011 ▸) the polarization factor takes the form 

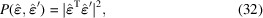

where 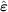 and 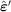 are the unit polarization vectors of the incident and scattered X-rays, respectively. An observer of an oscillating electron sitting on the scattered ray will only see oscillations that exist in the plane perpendicular to the propagation direction of the X-rays. Thus, we can describe 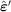 by the projection 

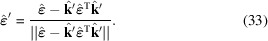

Inserting equation (33) in equation (32) we find 

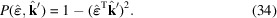




## 3. Software architecture

*xrd_simulator* is a Python library organized around four Python objects: an X-ray beam, a polycrystalline sample, a detector and a sample motion. These four Python objects are implementations of the mathematical concepts previously outlined in Section 2 and define together a diffraction experiment simulator. The end user of *xrd_simulator* can define their own simulations through Python scripts, instantiating each of the four necessary objects as desired. By passing a motion object to the polycrystalline sample, together with a beam and detector, diffraction vectors can be computed. Scattering units are computed and stored in the detector object. The user may then call a detector rendering method to compute a diffraction pattern image.

**Figure 6:**
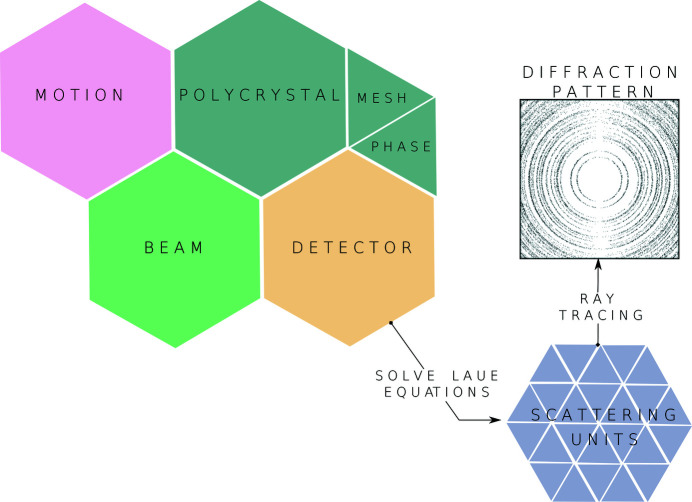
Four Python objects – an X-ray beam, a polycrystalline sample, a detector and a sample motion – define an experiment in *xrd_simulator*. Scattering units are computed and stored in the detector object. By selecting a ray tracing and intensity model a diffraction pattern image can be rendered.

A schematic overview of the *xrd_simulator* architecture can be found in Fig. 6 ▸. Detailed code samples and beginners tutorials on how to use *xrd_simulator* can be found both at GitHub (https://github.com/FABLE-3DXRD/xrd_simulator) as well as in the externally hosted documentation (https://fable-3dxrd.github.io/xrd_simulator/).

## 4. Software availability

The source code of *xrd_simulator* is openly distributed with an MIT open source licence at GitHub (https://github.com/FABLE-3DXRD/xrd_simulator). *xrd_simulator* features cross-platform support and can be installed using the Python package installer, *pip*, or alternatively the *Anaconda* package manager. Documentation on installing *xrd_simulator* can be found at the GitHub source location or, alternatively, in the externally hosted documentation (https://fable-3dxrd.github.io/xrd_simulator/).

## 5. Computational tractability

**Figure 7:**
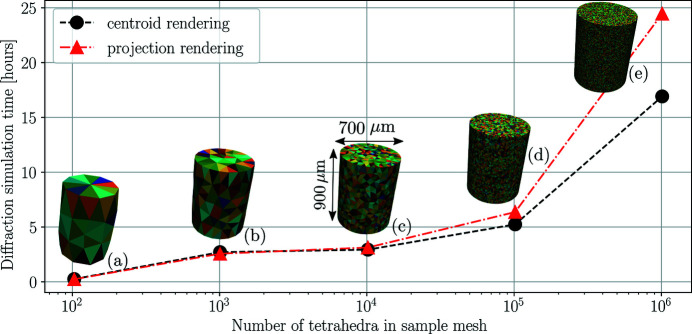
Typical compute times of *xrd_simulator* for a 10 ×10  × 180 × 2048 × 2048 pencil beam raster scan simulation. Diffraction was simulated from samples with random crystal orientations [coloured by one of their Bunge Euler angles in (*a*)–(*e*)]. For samples with many elements, a reduction in compute time is observed for the simplified ray tracing model described in Section 2.8.

The core computations of *xrd_simulator* can be summarized in three steps. Firstly, solutions to equation (17) are established. Secondly, polyhedral intersection regions between the X-ray beam and mesh elements are computed. Thirdly the diffraction signal is rendered into a diffraction pattern image. The total time needed to compute a diffraction pattern therefore scales with the number of elements within the mesh, the beam cross section and the angular range of the sample rotation. To enable computation of state-of-the-art data sets *xrd_simulator* implements a multiprocessing option using the Python native multiprocessing library. In Fig. 7 ▸ we provide some typical run times of *xrd_simulator* simulating a 10 × 10 pencil beam raster scan with 180 rendered frames in intervals of 1.0°. Considering the selected detector dimensions (2048  ×  2048) the computed data consisted of, in total, 10 × 10 × 180 × 2048 × 2048 ∼ 10^11^ floating point numbers. The timings presented in Fig. 7 ▸ were achieved on a Lenovo ThinkStation P330 MT deploying six Intel Core i7-8700K 3.70 GHz CPUs.

In conclusion, diffraction computations from samples with up to ∼10^6^ elements are feasible with *xrd_simulator* within 25 or 17 h, depending on what ray tracing model is selected.

## 6. Conclusions

An open source Python package for simulation of X-ray diffraction by polycrystals, named *xrd_simulator*, has been developed. By representing a polycrystalline sample as a tetrahedral mesh, an arbitrary sample morphology and microstructure can be modelled. Diffraction vectors are computed from the solutions of a time-dependent version of the Laue equations, enabling arbitrary rigid body motions of the sample. Diffraction peak intensities are computed as the product of scattering volumes and Lorentz, structure and polarization factors. Combining these features, *xrd_simulator* presents new opportunities to develop and understand the impact of different acquisition schemes for 3DXRD-type experiments such that optimal schemes can be defined in terms of acquisition time and resolution of the target parameters.

## Figures and Tables

**Figure 8:**
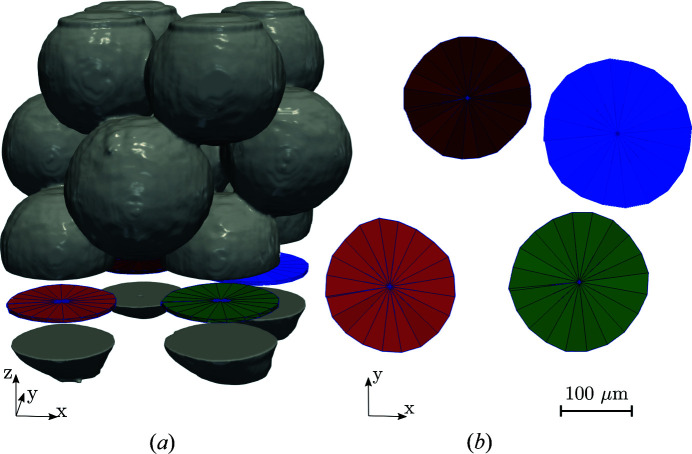
Exploded view of 12 α-quartz grains measured at the ESRF ID11 beamline (*a*). A single 20 µm-thick slice featuring four distinct grains was extracted and considered for simulation (*b*).

**Figure 9:**
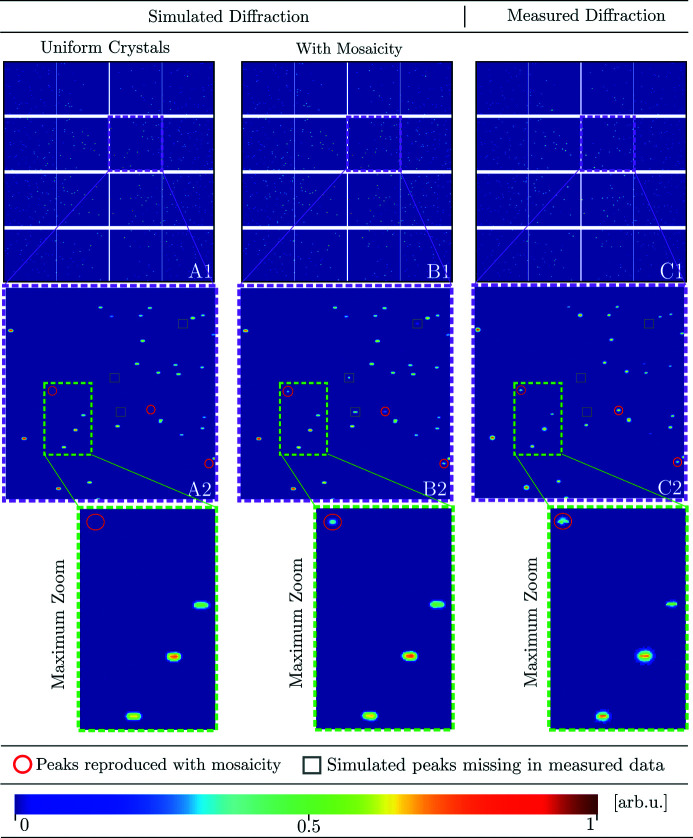
Simulated (A, B) and measured (C) log-normalized diffraction patterns from four 20 µm-thick 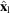 – 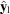 grain slices of α-quartz (SiO_2_). The diffraction pattern was integrated over a 10° sample rotation interval and is displayed with increasing levels of magnification in columns A, B and C, with the full tiled detector depicted in A1, B1 and C1. Diffraction peaks present in the true measured data which are only captured after the introduction of a random mosaicity are marked with circles. Diffraction peaks present in the simulated data but missing in the measurements are marked with squares.

**Figure 10:**
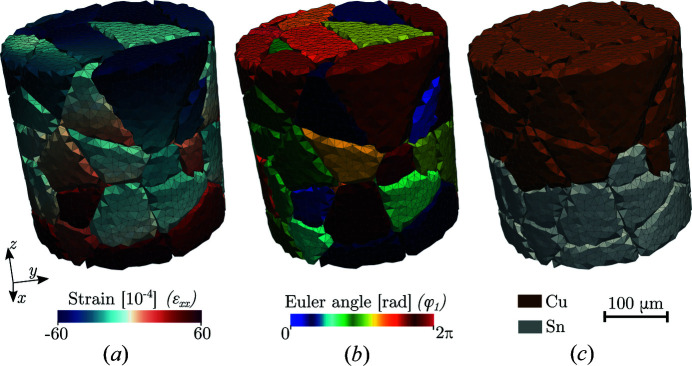
Phantom polycrystalline Cu–Sn aggregate composed of 120282 tetrahedral elements: (*a*) strain *xx* component, (*b*) Bunge Euler angle φ_1_ and (*c*) Cu–Sn phase map.

**Figure 11:**
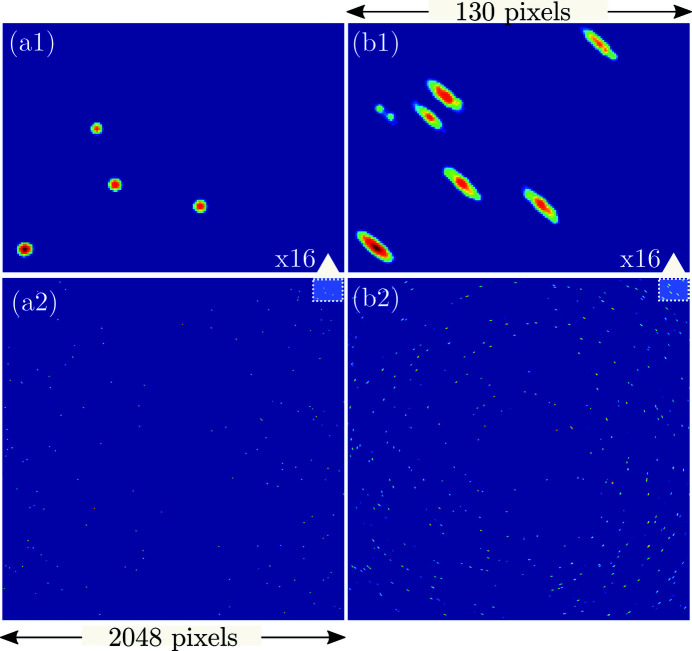
Simulated diffraction pattern from the phantom sample depicted in Fig. 10. Column (*a*) contains diffraction from an undeformed sample while column (*b*) depicts diffraction from a deformed version of the phantom. As a result, the diffraction peaks in the zoomed in area (*b*1) are distorted compared with the round diffraction peaks in (*a*1).

**Figure 12:**
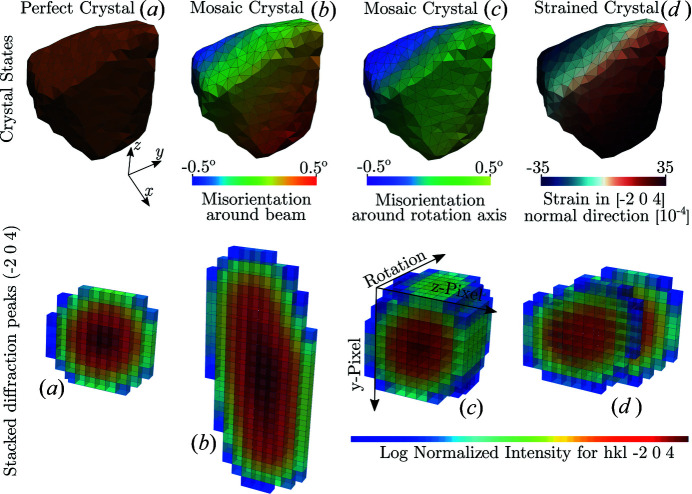
Single copper (Cu) grain extracted from the phantom in Fig. 10 composed of 4195 tetrahedral elements. The top row depicts induced deformation states while the bottom row shows the corresponding 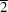 04 reflection rendered as a 3D peak, with sample rotational position as the third dimension.

**Table 1:** Simulation parameters used to render the diffraction patterns in Fig. 11 using the Cu–Sn phantom depicted in Fig. 10

Detector distance (µm)	191023.9164
Detector centre pixel *z*	1024.2345
Detector centre pixel *y*	1023.1129
Pixel side length *z* (µm)	50.4234
Pixel side length *y* (µm)	48.2343
Number of detector pixels *z*	2048
Number of detector pixels *y*	2048
Wavelength (Å)	0.18
Beam side length *z* (µm)	400
Beam side length *y* (µm)	400
Rotation step (1.0°)	1.0
Rotation axis	[0 0 1]

## Appendix A Experimental verification

To demonstrate the use of *xrd_simulator* we have simulated diffraction on the basis of measurements performed at the ESRF ID11 beamline. By comparing the results of *xrd_simulator* with the data from the experiment we explore the diffraction model limitations. The measured sample consisted of 12 quasi-spherical silica (SiO_2_) grains confined within a cylindrical polyether ether ketone tube and subject to 20 N of uni-axial loading along the laboratory *z*-axis direction. The full 3D grain volume was scanned with a scanning 3DXRD geometry (Hayashi *et al.*, 2015 ▸) first using a 20 µm × 20 µm pencil beam and then a 20 µm-height letter-box beam (covering the full sample in the  –  plane). The data from the pencil beam scan were used to perform tomographic reconstruction of the grain shapes, using a method similar to that reported by Poulsen & Schmidt (2003 ▸) [Fig. 8 ▸(*a*)]. The same pencil beam scan data were used to derive average crystallographic orientations and strain tensors of individual grains in the volume, using methods from *ImageD11* (Wright, 2005 ▸). A 20 µm-high slice (in the  –  plane) was extracted from the 3D tomographic reconstruction, to provide an equivalent volume to one of the 20 µm letterbox 3DXRD acquisitions. This volume was used to derive a grain mesh that was input to *xrd_simulator* [Fig. 8 ▸(*b*)] together with a crystallographic information file corresponding to α-quartz. In this way the input microstructure for *xrd_simulator* was derived soley from the pencil beam scan data while any of the following comparisons between simulated and measured diffraction patterns are made with the independently measured letterbox beam data.

Diffraction was simulated for the 20 µm-high slice through the sample by integrating the diffraction signal over a 10° rotation. The resulting 2D diffraction patterns were log-normalized and compared with the corresponding measured log-normalized signal (Fig. 9 ▸) from an equivalent letterbox acquisition.

Visual comparison between columns A and C in Fig. 9 ▸ shows similar diffraction patterns. However, the subset of diffraction peaks in Figs. 9 ▸-A2 and 9 ▸-C2 show some discrepancy between simulated and measured peak shapes. This is not unexpected and several potential sources of errors can be listed. These include:

(1) *Unknown detector point-spread function* will influence the peak shapes and maximal peak intensities.

(2) *Low signal-to-noise ratio* will influence low intensity scattering regions, which can lead to the removal of peaks or distortion of peak boundaries during background subtraction.

(3) *Unknown mosaicity and intragranular strain* will affect which peaks appear or not, as well as the peak shapes and intensities.

Turning our attention to the last of these error sources (3) we expect some, unknown, intragranular strain and orientation variations to be present within the individual grains. As a result the diffraction peak shapes will be deformed. Additionally, the set of possibly diffracting lattice plane families will be modified as the Bragg condition is shifted. To demonstrate these effects, modest, uniformly random mosaicity and strain variation were introduced into the simulation (Fig. 9 ▸ column B). First, each mesh element was seeded with the corresponding reconstructed grain average orientation matrix (derived from the pencil beam scan data). Next, the seeded orientation matrix was perturbed by a uniformly random rotation in the range 0.0–0.125°. Likewise, each component of strain was uniformly perturbed in the range 0–0.005. The magnitudes of the perturbations were chosen arbitrarily.

With the inclusion of random intragranular variations, we observe new, additional, diffraction peaks in Fig. 9 ▸-C as compared to Fig. 9 ▸-A. Although some diffraction events will now inevitably be erroneously pushed into their favourable Bragg conditions (marked with white squares in Figs. 9 ▸-A2, 9 ▸-B2 and 9 ▸-C2) several diffraction peaks originally missing in the simulation are now recovered (as marked with red circles in Figs. 9 ▸-A2, 9 ▸-B2 and 9 ▸-C2). This serves to illustrate how *xrd_simulator* captures the strong dependence between the measured diffraction signal and the underlying sample microstructure present in these types of experiments.

## Appendix B Highlights of software capability

*xrd_simulator* features space filling descriptions of polycrystals where each element of the tetrahedral mesh can have an individual phase, strain tensor and lattice orientation. To show how the spatial variation in a polycrystal can impact simulated diffraction patterns we provide far-field diffraction simulations from a multi-phase deformed polycrystal (Fig. 10 ▸) in this appendix section. As depicted in Fig. 10 ▸(*c*), a copper (Cu)–tin (Sn) aggregate composed of 64 grains with a combined total of 120282 individual tetrahedrons is considered. The individual grains were each seeded with a mean strain tensor and orientation matrix over which linear gradients in random directions were superimposed [Figs. 10 ▸(*a*) and 10 ▸(*b*)]. The aggregate was considered to be fully illuminated by 68.88 keV X-rays propagating along the *x* axis while the sample was rocked 1.0° around the *z* axis. To highlight the impact of the spatial deformation of the polycrystal, diffraction was simulated both with and without the prescribed strain and misorientations. The two resulting 2048 × 2048 pixelated diffraction patterns originating from a deformed and an undeformed sample can be viewed in Figs. 11 ▸(*a*2) and 11 ▸(*b*2), respectively. As depicted in Figs. 11 ▸(*a*1) and 11 ▸(*b*1) the impact of the lattice spatial variation is evident in the distorted diffraction peaks. Details of the experimental setup are presented in Table 1 ▸.

*xrd_simulator* offers a means to understand how the diffraction peak distortions relate to the internal grain deformation. To provide an example of how this can be utilized we have considered diffraction from a single Cu grain in the polycrystalline ensemble. The result of introducing a misorientation gradient around the beam direction and the axis of rotation are depicted in Figs. 12 ▸(*b*) and 12 ▸(*c*), respectively, where the resulting 3D peak shapes for the  04 reflection have been rendered. Likewise, the effect of a strain gradient in the (  04) crystal planes is depicted in Fig. 12 ▸(*d*). Comparing with a perfect crystal state [Fig. 12 ▸(*a*)], we see how the diffraction peak arcs over the detector for a misorientation around the beam axis while a misorientation around the rotation axis extends the angular range of diffraction. Finally, we can see the effect of a strain gradient in Fig. 12 ▸(*d*) resulting in a radially broadened and angularly extended diffraction peak.
